# Cancer

**Published:** 1890-01-25

**Authors:** 


### CANCER.

The arrest of growth of cancer by the interrupted voltaic current is the title of a paper published in the *Lancet* of November 30, by Dr. J. Inglis Parsons, assistant physician to the Chelsea Hospital for Women. The treatment is based on the fact that all the cells of the body possess a latent capability, which enables them under various stimuli to proliferate and form new tissue. It is possible that all tumours are, in the first instance, composed of cells formed in the normal process of repair, but that having escaped from the control of the nervous system, they take up an independent life. The frequent association of cancer with a depressed condition of the nervous system is explained by this hypothesis. Cancer of the breast is very frequently preceded by a history of inflammation or of injury. Tumours, although possessing vascular arrangements, are devoid of nerves. If a cure is found for cancer, it seems probable it may be based on the essential difference between tumour and normal tissue. And some agent should be found capable of inflicting an irreparable damage to a tumour and yet stop short of destroying normal cells. After several experiments, Dr. Parsons had no hesitation in trying the destructive effect of a powerful interrupted voltaic current in the first case of cancer that objected to be operated upon in the usual way. The *modus operandi* is as follows: The patient is anæsthetised. The current is then passed through the tumour and all the tissues for some inches round it by means of fine insulated needles, so as not to injure the skin. A battery of seventy cells, with an electromotive force of 105 volts, is used; the intensity of the current to commence with is ten milliamperes, gradually increased to 600 milliamperes, and flashed through the growth in every direction from fifty to one hundred times. The effects produced consist in a cessation of growth, gradual decrease of pain, shrinking and hardening of the tumour, followed by improved nutrition and a better standard of general health. The growth does not disappear, but remains an inert mass. One out of the four cases treated, having a presystolic bruit, was unable to stand more than 250 milliamperes. The advantages Dr. Parsons claims for this method are, that there is no destruction of normal tissue; if recurrence of growth occurs it can be stopped by repetition of the treatment; patients are not obliged to lie up for more than a day or two; they lose no blood; the current can be passed through almost any part of the body, and thus growths may be arrested which could not otherwise be treated. The earliest pathologists regarded cancer as a dyscrasia or altered condition of the blood, then followed the embryonic cell theory, finally the microbe theory. If the latter's theory of the causation of cancerous formations be correct, it may be understood that a voltaic current destroys the vitality of the microbe. However this may be, Dr. Parsons appears to have demonstrated that cancerous growths may be stayed by the voltaic current. Doubtless the treatment will be pursued, and the success already attained warrants the expectation that we have now a powerful means of at least arresting the formation of cancerous tissue.

In a further communication, Dr. Parsons states: "By comparing the action of the constant current on cancer with that of the interrupted voltaic current, I find that the former produces a destruction of tissues both healthy and morbid close to the poles, while the cells in the interpolar region retain their vitality; that the interrupted voltaic current apparently causes atrophy of morbid cells from pole to pole in the path of the cancer."

While on the subject of cancer we mention a communication to the *British Medical Journal* of December 7 from Mr. J. Brendon Curgenven, of Bayswater, on eucalyptus in cancers. In a case of cancer the odour was most sickening and offensive, apparently made worse by carbolic acid. The latter was stopped, and the eucalyptus disinfectant used. All offensive smell at once ceased.

